# Controlled Ovarian Stimulation Outcomes Across Different Malignancies in Women Undergoing Fertility Preservation: The Role of AMH and AFC in Predicting Ovarian Response

**DOI:** 10.3390/jcm15135144

**Published:** 2026-07-01

**Authors:** Loris Marin, Eleonora Targhetta, Federica Esposito, Guido Ambrosini, Alessandra Andrisani

**Affiliations:** Department of Women’s and Children’s Health, University of Padua, Via Giustiniani 3, 35128 Padua, Italy; eleonora.targhetta@studenti.unipd.it (E.T.); federica.esposito@aopd.veneto.it (F.E.); guido.ambrosini@unipd.it (G.A.); alessandra.andrisani@unipd.it (A.A.)

**Keywords:** fertility preservation, controlled ovarian stimulation, anti-Müllerian hormone, antral follicle count, oocyte cryopreservation

## Abstract

**Background/Objectives**: Fertility preservation is a key component of cancer care in women of reproductive age, and controlled ovarian stimulation (COS) followed by oocyte/embryo cryopreservation is considered the standard strategy. Although COS protocols are generally standardized, systemic effects related to different malignancies may interfere with ovarian reserve assessment and influence stimulation planning and counselling. This study aimed to evaluate COS outcomes across different malignancy types in women undergoing fertility preservation. **Methods**: This retrospective study included 331 oncological patients who underwent COS for fertility preservation at a single referral center between May 2008 and May 2025. Patients were stratified into three groups according to malignancy type: breast cancer, hematological malignancies, and other cancers. Ovarian reserve was assessed using serum anti-Müllerian hormone (AMH) levels and antral follicle count (AFC). COS outcomes, including stimulation duration, total gonadotropin dose, number of retrieved and mature oocytes, follicular output rate (FORT), and follicle-to-oocyte index (FOI), were compared among groups. **Results**: Patients with hematological malignancies were significantly younger and showed higher AFC values compared with the other groups, whereas AMH levels did not differ significantly. After adjustment for age and AFC, patients with hematological malignancies showed significantly lower AMH levels compared with breast cancer patients (β = −0.94 ng/mL, 95% CI −1.40 to −0.48, *p* < 0.001), supporting the presence of a relative AMH–AFC discordance. In unadjusted analyses, gonadotropin requirements were higher in breast cancer patients, whereas the number of retrieved and mature oocytes was higher in patients with hematological malignancies. However, after adjustment for age and AFC, malignancy type was not independently associated with retrieved oocytes, mature oocytes, total gonadotropin dose, or stimulation duration. FORT and FOI were comparable among groups. **Conclusions**: Malignancy type did not appear to independently impair ovarian responsiveness to stimulation. Patients with hematological malignancies showed preserved stimulation outcomes despite AMH values not being proportionally higher than expected based on their younger age and higher AFC. These findings suggest that AMH should be interpreted with caution in systemic malignancies and that AFC may be particularly useful for tailoring fertility preservation counselling and stimulation strategies when AMH and AFC are discordant.

## 1. Introduction

Hematological malignancies represent the most frequently diagnosed cancers in prepubertal age, with acute lymphoblastic leukemia (ALL) being the most common, followed by brain and central nervous system tumors [1,2]. During adolescence, the most prevalent malignancies include Hodgkin lymphoma, non-Hodgkin lymphoma, and leukemias, in particular acute myeloblastic leukemia [2]. In adult women, breast cancer is the most commonly diagnosed malignancy [2,3]. Due to advances in early diagnosis and oncological treatments, survival rates among cancer patients have markedly improved over recent decades [3]. As a consequence, survivorship is associated with new challenges, particularly regarding long-term quality of life and reproductive health [4,5]. For women diagnosed with cancer during their reproductive years, the risk of treatment-induced infertility represents a major concern. Chemotherapy, radiotherapy, and, in some cases, surgical interventions may exert gonadotoxic effects, compromising ovarian reserve and reproductive potential [4,5].

This issue is further compounded by sociocultural trends toward delayed childbearing [6], resulting in a growing number of women receiving a cancer diagnosis before completing their reproductive plans [5,7]. Consequently, fertility preservation has become an integral component of cancer care in young women [8,9]. Controlled ovarian stimulation (COS) followed by oocyte or embryo cryopreservation is considered the gold standard strategy for fertility preservation in post-pubertal women [8,9]. While COS protocols are largely standardized and drug dosing is generally determined by age and ovarian reserve parameters, cancer heterogeneity may influence ovarian response. In particular, hormone-sensitive tumors, such as certain subtypes of breast cancers, require the concomitant administration of aromatase inhibitors during COS to prevent excessive estrogen increase [10,11]. Apart from this specific setting, COS protocols are usually applied irrespective of tumor type. Nevertheless, it is known that different malignancies may exert distinct systemic effects. Hematological malignancies, for example, are characterized by high levels of circulating cytokines, which are known to play a role in folliculogenesis and oocyte maturation [12,13,14]. These disease-related factors may potentially influence ovarian responsiveness to stimulation. The aim of this study was to evaluate COS outcomes across different types of malignancies in women undergoing fertility preservation and to assess the role of AMH and AFC in predicting ovarian response. We hypothesized that malignancy type would not independently impair ovarian responsiveness to stimulation after accounting for age and ovarian reserve.

## 2. Materials and Methods

### 2.1. Study Design and Population

This retrospective cohort study included 331 female cancer patients who underwent COS for fertility preservation between May 2008 and May 2025 at the IVF Center of Padua Hospital (Department of Women’s and Children’s Health, University of Padua, Italy). Patients were excluded if they had previously undergone ovarian surgery, gonadotoxic chemotherapy, pelvic radiotherapy, or any other treatment potentially affecting ovarian reserve before fertility preservation. Patients were divided into three groups according to malignancy type: breast cancer (Group A), hematological malignancies (Group B), and other types of cancer (Group C).

This retrospective study was approved by the Ethics Committee of the University Hospital of Padua (protocol number 6478n/AO/25; approval date: 13 November 2025). The approval referred to retrospective access to clinical data and their use for research purposes. All fertility preservation procedures had been performed as part of standard clinical care, and procedure-specific informed consent was obtained before each intervention. Patients were subsequently contacted, and written informed consent was obtained for the use of anonymized clinical data for the present study and for publication purposes.

### 2.2. Ovarian Reserve Assessment

At the first oncofertility consultation, all patients underwent biochemical and ultrasonographic assessment of ovarian reserve before starting COS. Serum anti-Müllerian hormone (AMH) levels and antral follicle count (AFC) were recorded. AMH measurements were performed in the same laboratory. Until 24 May 2019, AMH was measured using a conventional enzyme-linked immunosorbent assay (Beckman Coulter, Milan, Italy). From 24 May 2019 onward, AMH was measured using an electrochemiluminescence immunoassay (Elecsys^®^ AMH Plus, Roche Diagnostics, Mannheim, Germany). Because the assay principle and reference intervals changed with the transition to the new method, no formal overlap study or conversion factor was applied between the two assays. Transvaginal ultrasound examinations were performed by skilled operators using high-resolution ultrasound machines (Voluson E6, GE HealthCare, Zipf, Austria; and Hera W10, Samsung Medison Co., Ltd., Hongcheon-gun, Republic of Korea) equipped with endocavitary transducers: Voluson E6 with a RIC5-9-D 4–9 MHz endocavitary transducer (GE HealthCare, Zipf, Austria) and HERA W10 with an EV2-10A 2–10 MHz endocavitary transducer (Samsung Medison Co., Ltd., Hongcheon-gun, Republic of Korea).

### 2.3. Stimulation Protocols

All women underwent either a short GnRH-antagonist protocol or a progestin-primed ovarian stimulation protocol (PPOS), with either random- or conventional-start, according to the menstrual cycle phase at the time of first consultation. Follitropin was administered to all patients, with the starting dose individualized according to ovarian reserve parameters. In case of discordance between AMH and AFC, AFC was used as the primary predictor of oocyte yield [15]. In patients with hormone-sensitive tumors, letrozole was co-administered in accordance with international guidelines.

### 2.4. Outcomes

The primary outcomes of the study were the comparison among the three groups of the total number of retrieved oocytes, number of mature (MII) oocytes, total gonadotropin dose and COS duration. Secondary outcomes assessed between groups were follicular output rate (FORT) and follicle-to-oocyte index (FOI). FORT was calculated as the ratio between the number of follicles > 14 mm on the day of ovulation induction and the antral follicle count (AFC), while FOI was calculated as the ratio between retrieved oocytes and follicles > 14 mm.

### 2.5. Statistical Analysis

Statistical analysis was performed using SPSS version 22.0 software (SPSS Inc, Chicago, IL, USA). Continuous variables were reported as mean ± standard deviation, whereas categorical variables were presented as absolute frequencies and percentages. Student’s *t*-test or Mann–Whitney U test was used for comparisons of continuous variables, as appropriate. Categorical variables were compared using chi-square test or Fisher’s exact test. Multivariable linear regression analyses were performed to evaluate whether malignancy type was independently associated with COS outcomes. Separate models were built for retrieved oocytes, mature MII oocytes, total gonadotropin dose, and stimulation duration. Malignancy group was included as the main independent variable, with breast cancer used as the reference category. First, age-adjusted models were performed. Subsequently, models additionally adjusted for AFC were used to assess whether malignancy type was associated with ovarian stimulation outcomes independently of the recruitable antral follicle pool. Sensitivity analyses including AMH were performed in the subset of patients with available AMH values. To further investigate the presence of a relative AMH–AFC discordance, AMH was used as the dependent variable in a multivariable linear regression model including malignancy group, age, and AFC as independent variables. Results from regression models were reported as unstandardized beta coefficients (β), 95% confidence intervals (CI), and *p* values. A *p* value < 0.05 was considered statistically significant.

## 3. Results

Among the 331 patients included in the study, 178 had breast cancer (Group A, 53.8%), 88 had hematological malignancies, predominantly Hodgkin and non-Hodgkin lymphoma (Group B, 26.6%) and 65 had other malignancies (Group C, 19.6%). Group C included a heterogeneous spectrum of malignancies. The most frequent primary tumor sites were the central nervous system (20/65, 30.8%), ovary/adnexa (9/65, 13.8%), bone and soft tissue sarcomas (8/65, 12.3%), and gastrointestinal tract/appendix (7/65, 10.8%) (Supplementary Table S1).

The mean age of the overall study population was 30.4 ± 5.8 years, with an age range of 18–45. Age differed significantly among the three groups (*p* < 0.001). Patients with breast cancer were the oldest with a mean age of 32.6 ± 4.7 years, followed by patients with other malignancies, 29.2 ± 5.7 years, and patients with hematological malignancies, 26.2 ± 5.4 years.

Mean BMI was within the normal range in all groups, with an overall mean value of 22.0 ± 4.37 kg/m^2^. Mean BMI was 21.4 ± 3.9 in group A, 23.0 ± 5 in group B, and 22.3 ± 4.5 in group C. A significant difference was observed only between group A and group B (*p* = 0.003).

**Figure 1:**
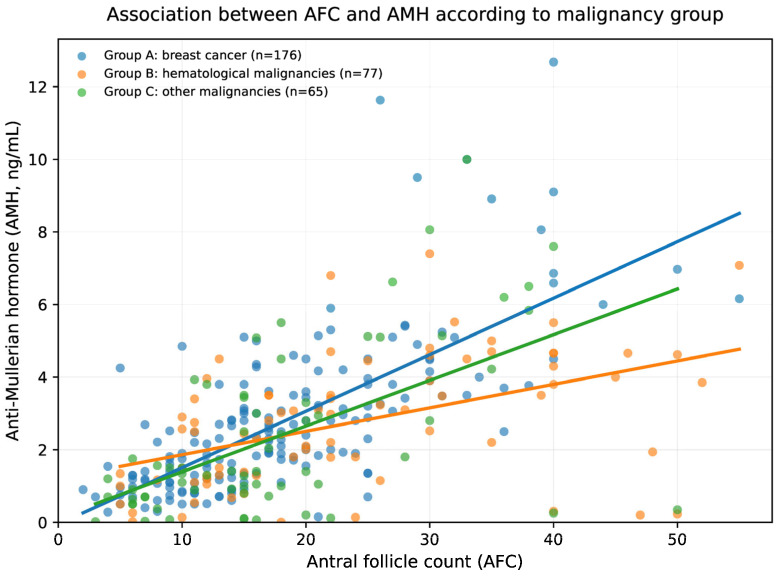
Association between AFC and AMH according to malignancy group. Scatterplot showing the relationship between antral follicle count and serum AMH levels across malignancy groups. Dots represent individual patients and lines represent group-specific ordinary least squares regression lines. Line colors correspond to the malignancy groups shown in the figure legend. Group A: breast cancer; Group B: hematological malignancies; Group C: other malignancies.

Baseline ovarian reserve markers differed across groups. Group B showed significantly higher AFC values compared with the other groups, with a mean AFC of 23.5 ± 13.0, compared with 18.2 ± 9.6 in Group A and 17.8 ± 10.4 in group C (*p* < 0.001). AMH values were unavailable for a subset of patients due to missing records. AMH measurements were available for 176 women in Group A, 77 in Group B, and all women in Group C. Mean AMH levels were 2.61 ± 2.4 ng/mL in Group A, 2.75 ± 1.8 ng/mL in Group B, and 2.37 ± 2.3 ng/mL in Group C. No statistically significant differences were observed in pairwise comparisons between groups: Group A versus Group B, *p* = 0.65; Group A versus Group C, *p* = 0.48; Group B versus Group C, *p* = 0.27 (Table 1). To further investigate the apparent discrepancy between higher AFC values and comparable AMH levels in Group B, AMH was used as the dependent variable in a multivariable linear regression model including malignancy group, age, and AFC as covariates. After adjustment for age and AFC, patients with hematological malignancies showed significantly lower AMH levels compared with patients with breast cancer (β = −0.94 ng/mL, 95% CI −1.40 to −0.48, *p* < 0.001). In contrast, the difference between patients with other malignancies and those with breast cancer did not reach statistical significance (β = −0.52 ng/mL, 95% CI −1.07 to 0.03, *p* = 0.064). These findings support the presence of a relative AMH–AFC discordance in patients with hematological malignancies. The relationship between AFC and AMH according to malignancy group is shown in Figure 1.

Regarding stimulation protocol, 214 patients, corresponding to 64.65% of the overall cohort, underwent random-start stimulation, while 117 patients, corresponding to 35.35%, underwent conventional-start stimulation, defined as COS initiation within the fourth day of the menstrual cycle. The distribution of random-start and conventional-start protocols was comparable among the three groups. Specifically, random-start stimulation was performed in 118 patients with breast cancer (66.3%), 57 patients with hematological malignancies (64.8%), and 39 with other malignancies (60.0%). No statistically significant differences in protocol distribution were observed across groups (Table 1).

The mean duration of ovarian stimulation was 11.3 ± 2.1 days in group A, 11.2 ± 2.0 days in group B, and 10.7 ± 2.4 days in Group C, with no statistically significant differences among groups. The mean total gonadotropin dose administered during stimulation was 3217.7 ± 1330.2 IU in Group A, 2630.9 ± 1100.7 IU in group B, and it was 3148.9 ± 1304.6 IU in group C. In unadjusted analyses, statistically significant differences in gonadotropin dose were observed between Group A and Group B (*p* = 0.0004) and between Group B and Group C (*p* = 0.0087).

The mean number of retrieved oocytes was 14.5 ± 9.4 in group A, 17.5 ± 10.7 in group B, and 14.8 ± 10.7 in group C. In unadjusted analyses, a statistically significant difference was observed only between Groups A and B (*p* = 0.02). Similarly, the mean number of mature oocytes was 11.3 ± 7.3 in group A, 13.7 ± 8.4 in group B, and 11.8 ± 8.3 in group C, with a statistically significant difference observed only between Group A and B (*p* = 0.016). Unadjusted COS outcomes are reported in Table 2. To account for differences in age and ovarian reserve among groups, multivariable regression analyses were performed and are reported in Table 3.

Data are presented as mean ± standard deviation. Reported *p* values refer to unadjusted comparisons between groups. For outcomes with significant differences, *p* values refer to pairwise comparisons. Multivariable adjusted analyses are reported in Table 3.

**Figure 2:**
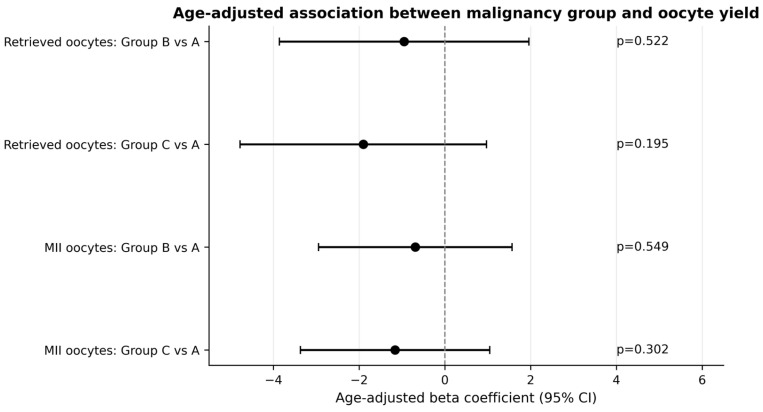
Age-adjusted association between malignancy group and oocyte yield. Forest plot showing beta coefficients and 95% confidence intervals from multivariable linear regression models adjusted for age. Black dots represent beta coefficients, horizontal lines represent 95% confidence intervals, and the dashed vertical line indicates no difference compared with the reference group. Breast cancer was used as the reference category.

To determine whether these differences were independent of age, age-adjusted multivariable linear regression analyses were performed (Figure 2).

After adjustment for age, malignancy group was no longer significantly associated with either the number of retrieved oocytes or the number of mature oocytes. Compared with patients with breast cancer, patients with hematological malignancies did not show a significantly different number of retrieved oocytes (β = −0.95, 95% CI −3.87 to 1.97, *p* = 0.523) or mature oocytes (β = −0.69, 95% CI −2.96 to 1.58, *p* = 0.549). Similarly, no significant differences were observed between patients with other malignancies and those with breast cancer for retrieved oocytes (β = −1.90, 95% CI −4.79 to 0.98, *p* = 0.195) or mature oocytes (β = −1.16, 95% CI −3.38 to 1.05, *p* = 0.303). Age was independently and negatively associated with both retrieved oocytes (β = −0.59, 95% CI −0.81 to −0.37, *p* < 0.001) and mature oocytes (β = −0.46, 95% CI −0.64 to −0.29, *p* < 0.001).

Additional multivariable models adjusted for both age and AFC were then performed to evaluate whether malignancy group influenced ovarian response independently of the recruitable follicular pool. In these models, malignancy group remained non-significantly associated with both retrieved and mature oocyte yield. Compared with breast cancer patients, patients with hematological malignancies did not show a significantly different number of retrieved oocytes (β = −1.58, 95% CI −3.88 to 0.72, *p* = 0.177) or mature oocytes (β = −1.17, 95% CI −2.97 to 0.63, *p* = 0.202). Patients with other malignancies also showed no significant differences compared with breast cancer patients in terms of retrieved oocytes (β = −0.42, 95% CI −2.58 to 1.73, *p* = 0.699) or mature oocytes (β = −0.04, 95% CI −1.71 to 1.63, *p* = 0.962). In contrast, AFC remained independently associated with both retrieved oocytes (β = 0.54, 95% CI 0.44 to 0.64, *p* < 0.001) and mature oocytes (β = 0.41, 95% CI 0.34 to 0.48, *p* < 0.001) (Table 3).

Age- and AFC-adjusted models were also performed for total gonadotropin dose and stimulation duration. Malignancy group was not independently associated with either total gonadotropin dose or stimulation duration. For total gonadotropin dose, neither hematological malignancies (β = 38.10, 95% CI −257.75 to 333.96, *p* = 0.800) nor other malignancies (β = 83.76, 95% CI −229.17 to 396.70, *p* = 0.599) differed significantly from breast cancer. Age was positively associated with total gonadotropin dose (β = 46.75, 95% CI 23.88 to 69.62, *p* < 0.001), whereas AFC was negatively associated with total gonadotropin dose (β = −58.08, 95% CI −68.77 to −47.39, *p* < 0.001) (Table 3). Similarly, for stimulation duration, malignancy group was not independently associated with the outcome, whereas older age and lower AFC were associated with slightly longer stimulation duration (Table 3).

Sensitivity analyses including AMH were performed in the subset of patients with available AMH values. In models including age, AFC, AMH, and malignancy group, malignancy type was not independently associated with either retrieved or mature oocyte yield, whereas both AFC and AMH remained independent predictors of these outcomes. In subgroup analysis restricted to patients with hematological malignancies, AFC remained independently associated with mature oocyte yield (β = 0.36, *p* < 0.001), whereas AMH did not (β = 0.84, *p* = 0.139), suggesting that AFC may be particularly informative for predicting mature oocyte yield in this subgroup.

The mean FOI in the overall population was 0.86 ± 0.60. Mean FOI values were 0.87 ± 0.65 in group A, 0.83 ± 0.48 in group B, and 0.89 ± 0.60 in group C. No statistically significant differences were observed in any of the pairwise comparisons: group A vs. group B, *p* = 0.5636; group A vs. group C, *p* = 0.8880; group B vs. group C, *p* = 0.5055.

The mean FORT in the overall cohort was 0.75 ± 0.45. In subgroup analyses, mean FORT values were 0.78 ± 0.47 in Group A, 0.73 ± 0.37 in Group B, and 0.68 ± 0.54 in Group C. No statistically significant differences were observed in pairwise comparisons: group A vs. group B, *p* = 0.4162; group A vs. group C, *p* = 0.4921; group B vs. group C, *p* = 0.1707. Therefore, both FOI and FORT were analyzed as continuous variables, as categorical stratifications did not provide additional discriminatory value among malignancy groups (Table 2).

## 4. Discussion

The aim of this study was to evaluate COS outcomes for fertility preservation across distinct malignancy groups. In our cohort, breast cancer represented the most frequent diagnosis, in line with global cancer epidemiology. Patients with hematological malignancies were significantly younger than those in the other groups, which is consistent with epidemiological data showing that hematological malignancies, particularly lymphomas, tend to occur at a younger age compared with breast cancer [16]. Although BMI differed significantly among groups, the absolute difference was small and mean BMI values remained within the normal range in all groups. Therefore, this difference is unlikely to be biologically meaningful or to have substantially influenced ovarian stimulation outcomes.

Ovarian reserve was assessed using both AFC and serum AMH. AFC differed significantly among groups, with higher values in younger patients with hematological malignancies. This finding was expected, as ovarian reserve is strongly age-dependent [17,18]. In contrast, unadjusted AMH levels did not differ significantly among the three groups. Given the younger age and higher AFC of patients with hematological malignancies, proportionally higher AMH levels would have been expected [19,20]. The absence of such a difference suggests that AMH levels in women with hematological malignancies may already be relatively reduced prior to chemotherapy [21,22]. Available literature on ovarian reserve in patients with hematological malignancies is discordant. Lawrenz et al. [21] and Lekovich et al. [22] reported reductions in both AFC and AMH levels in patients with hematological malignancies, suggesting a cancer-related impairment of ovarian reserve, possibly due to disease-related systemic inflammation, cytokine-mediated granulosa cell dysfunction, or altered follicular AMH secretion rather than true depletion of the primordial follicle pool. Conversely, Su et al. [23] described AMH reduction also in breast cancer patients, while other studies reported no significant differences in AMH levels between cancer patients and healthy controls [24,25]. Dolinko et al. [26] observed reduced baseline AFC and increased gonadotropin requirements in patients with systemic malignancies. These conflicting findings highlight the lack of consensus regarding the impact of cancer itself on ovarian reserve markers.

In our study, patients with hematological malignancies showed significantly higher AFC values compared with the other groups, while unadjusted AMH levels were not significantly different. However, after adjustment for age and AFC, AMH levels were significantly lower in patients with hematological malignancies compared with breast cancer patients. This finding supports the presence of a relative AMH–AFC discordance in this subgroup and suggests that AMH may be influenced by disease-related factors beyond the size of the recruitable antral follicle pool. From a clinical perspective, an adjusted difference of approximately 1 ng/mL may be relevant when AMH is used in isolation for ovarian reserve counselling or gonadotropin dose selection. However, in our cohort, this lower-than-expected AMH was not accompanied by impaired stimulation outcomes. Therefore, since both AFC and AMH reflect the antral follicle compartment, an isolated relative reduction in AMH should not be automatically interpreted as evidence of diminished ovarian reserve, but should rather be interpreted together with AFC and the overall clinical context.

The mechanism underlying the relative AMH–AFC discordance observed in patients with hematological malignancies remains speculative. One possible explanation is that the systemic inflammatory milieu and cytokine network activation associated with lymphomas may impair granulosa cell function and reduce AMH secretion without necessarily reflecting true depletion of the recruitable antral follicle pool. This hypothesis is supported by studies attributing AMH suppression to cytokine-mediated effects. Paradisi et al. [14] demonstrated that cytokine network hyperactivity in both Hodgkin and non-Hodgkin lymphoma may inhibit AMH secretion, showing an inverse correlation between AMH levels and specific inflammatory cytokines [21,27]. Additionally, van Dorp et al. [28] suggested that the general health status of patients with systemic malignancies could impair AMH secretion independently of the primordial follicle pool, possibly through granulosa cell dysfunction. Whether cytokine-mediated AMH suppression is transient or reflects persistent granulosa cell dysfunction before chemotherapy remains unknown. However, the preserved FORT, FOI, and oocyte yield observed in our cohort suggest that the lower-than-expected AMH was not associated with clinically evident impairment of ovarian responsiveness at the time of stimulation. These findings are more consistent with a functional or secretory alteration of AMH than with irreversible damage to the recruitable follicular pool, although longitudinal studies are needed to clarify the reversibility and biological significance of this phenomenon.

The AMH–AFC discordance was less evident in breast cancer patients, possibly because breast cancer is generally less characterized by the systemic inflammatory and cytokine-mediated milieu typical of lymphomas [14,27]. Importantly, our findings do not suggest that low AMH values should be disregarded. Rather, when AMH is unexpectedly low despite preserved AFC, particularly in patients with systemic malignancies, AFC should be given particular weight for counselling and gonadotropin dose planning, while AMH should still be interpreted within the overall clinical context.

Other authors have reported discordant findings. Caringella et al. [29] described lower AMH levels with preserved AFC in patients with hematological malignancies; however, in contrast to our results, they also observed reduced fertility preservation outcomes, particularly in terms of mature oocytes retrieved. The discrepancy between our findings and those of Caringella et al. [29] may reflect differences in patient selection, hematological malignancy subtype distribution, disease burden, baseline ovarian reserve, stimulation protocols, and the timing of fertility preservation in relation to cancer diagnosis. Differences in statistical adjustment may also be relevant, since the apparent association between malignancy type and oocyte yield may be attenuated after accounting for age and AFC. Taken together, these findings suggest that although AMH–AFC discordance may be observed in patients with systemic malignancies, its clinical implications for fertility preservation outcomes are not uniform and should be interpreted with caution. In unadjusted analyses, patients with hematological malignancies showed a higher number of retrieved and mature oocytes compared with breast cancer patients, while breast cancer patients required higher gonadotropin doses. However, these differences were no longer independently associated with malignancy type after adjustment for age and AFC. Therefore, the higher oocyte yield observed in the hematological group was largely explained by younger age and higher AFC rather than by malignancy type itself. These findings support the notion that ovarian responsiveness to stimulation is preserved across malignancy groups.

Ovarian responsiveness to stimulation was further assessed using FORT and FOI, which reflect different biological phases of the stimulation process [30,31]. No clinically relevant differences suggesting impaired ovarian responsiveness were observed in FORT and FOI across malignancy groups. These findings indicate that follicular sensitivity to gonadotropins, follicular growth to advanced stages, oocyte maturation, and oocyte retrieval efficiency are preserved even in patients with systemic malignancies, including those with hematological malignancies. However, preserved FORT and FOI reflect ovarian responsiveness during stimulation and should not be interpreted as direct evidence of equivalent oocyte competence or reproductive potential. Data on oocyte utilization, fertilization, embryo development, pregnancy, and live birth rates were not available in the present study.

An important implication of these results is that AFC may deserve particular consideration in patients with systemic malignancies when discordance between AMH and AFC is observed. This concept is supported by previous evidence from Hochberg et al. [15], who emphasized the importance of prioritizing AFC when discordance between AMH and AFC is observed. Accurate evaluation of the ovarian reserve is crucial for fertility preservation counselling and for determining the appropriate starting dose of gonadotropins during COS.

This aspect is particularly relevant in oncology patients, who often have only one opportunity for ovarian stimulation before starting gonadotoxic treatment. Accurate assessment of ovarian reserve is therefore crucial not only to optimize stimulation strategies and maximize oocyte yield, but also to provide appropriate and realistic fertility preservation counselling. Relying solely on AFC may carry clinical risks, particularly in young patients with high AFC, in whom ovarian response may be overestimated or gonadotropin dosing may be inappropriate. Therefore, AFC should not be used in isolation but integrated with age, BMI, AMH, clinical urgency, previous ovarian history, and close monitoring during stimulation. This approach is essential to minimize the risk of ovarian hyperstimulation syndrome-related complications, which may delay the initiation of oncological treatment and potentially impact oncological prognosis [32].

The main strengths of this study include the relatively large cohort of cancer patients undergoing fertility preservation in a single tertiary referral center and the availability of both biochemical and ultrasonographic ovarian reserve markers. However, several limitations should be acknowledged. First, the retrospective design limits causal inference. Second, the long study period may have introduced changes in clinical practice, stimulation protocols, laboratory procedures, and AMH assays over time. In particular, although all AMH measurements were performed in the same laboratory, the transition from ELISA to electrochemiluminescence immunoassay during the study period may have introduced inter-assay variability. Third, the heterogeneous composition of Group C represents a limitation. This group included different tumor sites and biological entities, and this heterogeneity may have masked disease-specific variations in ovarian reserve markers and stimulation outcomes. Therefore, results for Group C should be interpreted as exploratory. In addition, the study was not powered to detect differences within specific hematological malignancy subtypes, such as Hodgkin versus non-Hodgkin lymphoma. Long-term reproductive outcomes, including oocyte utilization, fertilization, pregnancy, and live birth rates, were not available; therefore, we could not determine whether preserved ovarian responsiveness translated into equivalent reproductive outcomes across malignancy groups. Finally, although multivariable analyses were performed, residual confounding cannot be excluded.

## 5. Conclusions

Our findings suggest that fertility preservation outcomes are not directly influenced by malignancy type per se. In patients with hematological malignancies, AMH values may be relatively lower than expected based on age and AFC, supporting the presence of a relative AMH–AFC discordance. In this setting, AFC may be particularly useful for predicting ovarian response and guiding individualized stimulation strategies. Further prospective studies are needed to confirm these findings and to clarify the mechanisms underlying AMH suppression in systemic malignancies.

## Figures and Tables

**Table 1:** Baseline characteristics of the study population according to malignancy type.

	Group A (*n* = 178)	Group B (*n* = 88)	Group C (*n* = 65)	*p*-Value
Age (years)	32.6 ± 4.7	26.2 ± 5.4	29.2 ± 5.7	<0.001
BMI (kg/m^2^)	21.4 ± 3.9	23.0 ± 5.0	22.3 ± 4.5	0.003
AFC	18.2 ± 9.6	23.5 ± 13	17.8 ± 10.4	<0.001
AMH (ng/mL)	2.61 ± 2.4	2.75 ± 1.8	2.37 ± 2.3	NS
Random-start, *n* (%)	118 (66.3)	57 (64.8)	39 (60)	NS

**Table 2:** Descriptive comparison of controlled ovarian stimulation outcomes according to malignancy type.

	Group A	Group B	Group C	*p*-Value
Stimulation duration (days)	11.3 ± 2.1	11.2 ± 2.0	10.7 ± 2.4	NS
Total gonadotropin dose (IU)	3217.7 ± 1330.2	2630.9 ± 1100.7	3148.9 ± 1304.6	A vs. B: 0.0004 B vs. C: 0.0087
Retrieved oocytes	14.5 ± 9.4	17.5 ± 10.7	14.8 ± 10.7	A vs. B: 0.02
MII oocytes	11.3 ± 7.3	13.7 ± 8.4	11.8 ± 8.3	A vs. B: 0.016
FOI	0.87 ± 0.65	0.83 ± 0.48	0.89 ± 0.60	NS
FORT	0.78 ± 0.47	0.73 ± 0.37	0.68 ± 0.54	NS

NS: not significant.

**Table 3:** Age- and AFC-adjusted multivariable analysis of ovarian stimulation outcomes.

Retrieved Oocytes			
Variable	β Coefficient	95% CI	*p* Value
Hematological vs. breast cancer	−1.58	−3.88 to 0.72	0.177
Other cancers vs. breast cancer	−0.42	−2.58 to 1.73	0.699
Age	−0.25	−0.45 to −0.06	0.012
AFC	0.54	0.44 to 0.64	<0.001
MII oocytes			
Variable	β coefficient	95% CI	*p* value
Hematological vs. breast cancer	−1.17	−2.97 to 0.63	0.202
Other cancers vs. breast cancer	−0.04	−1.71 to 1.63	0.962
Age	−0.21	−0.36 to −0.06	0.007
AFC	0.41	0.34 to 0.48	<0.001
Total gonadotropin dose			
Variable	β coefficient	95% CI	*p* value
Hematological vs. breast cancer	38.10	−257.75 to 333.96	0.800
Other cancers vs. breast cancer	83.76	−229.17 to 396.70	0.599
Age	46.75	23.88 to 69.62	<0.001
AFC	−58.08	−68.77 to −47.39	<0.001
Stimulation duration			
Variable	β coefficient	95% CI	*p* value
Hematological vs. breast cancer	0.35	−0.30 to 1.00	0.292
Other cancers vs. breast cancer	−0.41	−1.08 to 0.27	0.235
Age	0.05	0.004 to 0.10	0.033
AFC	−0.03	−0.05 to −0.004	0.019

## Data Availability

The data presented in this study are available from the corresponding author upon reasonable request.

## Supplementary Materials

The following supporting information can be downloaded at: https://www.mdpi.com/article/10.3390/jcm15135144/s1, Table S1: Distribution of malignancies included in Group C according to primary tumor site. Data are presented as numbers and percentages. Percentages were calculated among patients included in Group C.

## Abbreviations

The following abbreviations are used in this manuscript:

AFC	Antral follicle count
AMH	Anti-Müllerian hormone
COS	Controlled ovarian stimulation
FORT	Follicular output rate
FOI	Follicle-to-oocyte index
MII	Metaphase II
PPOS	Progestin-primed ovarian stimulation
