# Supplementary material for: Controlled Ovarian Stimulation Outcomes Across Different Malignancies in Women Undergoing Fertility Preservation: The Role of AMH and AFC in Predicting Ovarian Response
Source: J Clin Med. 2026 Jul 1;15(13):5144. doi: 10.3390/jcm15135144 (PMC13362775; doi:10.3390/jcm15135144)
Supplement: Supplementary file 1 [file jcm-15-05144-s001.zip › jcm-4360388-supplementary.pdf]

Supplementary Table S1. Distribution of malignancies included in Group C according to primary tumor site. Data are presented as number and percentage. Percentages were calculated among patients included in Group C.

Primary tumor site	Diagnoses included	n (%)
Central nervous system	Astrocytoma, glioblastoma, medulloblastoma, oligodendroglioma	20 (30.8%)
Ovary	Borderline ovarian tumor, dysgerminoma, Brenner tumor	9 (13.8%)
Bone and soft tissue sarcomas	Desmoid tumor, osteosarcoma, rhabdomyosarcoma, Ewing sarcoma	8 (12.3%)
Gastrointestinal tract and appendix	Colorectal cancer, mucinous appendiceal neoplasm, pseudomyxoma peritonei, gastric neuroendocrine tumor	7 (10.8%)
Skin	Melanoma	6 (9.2%)
Head and neck	Tongue, oropharyngeal, nasopharyngeal and oral cavity cancers	5 (7.7%)
Thyroid	Thyroid cancer	5 (7.7%)
Pituitary gland	Pituitary tumors	2 (3.1%)
Adrenal gland	Pheochromocytoma	1 (1.5%)
Thymus	Thymoma	1 (1.5%)
Kidney	Wilms tumor	1 (1.5%)
**Total**		**65 (100%)**
